# Reproductive ecology and stand structure of Joshua tree forests across climate gradients of the Mojave Desert

**DOI:** 10.1371/journal.pone.0193248

**Published:** 2018-02-23

**Authors:** Samuel B. St. Clair, Joshua Hoines

**Affiliations:** 1 Department of Plant and Wildlife Sciences, Brigham Young University, Provo, Utah, United States of America; 2 Death Valley National Park, Division of Resources Management, Death Valley, California, United States of America; US Geological Survey, UNITED STATES

## Abstract

Climate change is restructuring plant populations and can result in range shifts depending on responses at various life stages of plants. In 2013, a widespread and episodic flowering event provided an opportunity to characterize how Joshua tree’s reproductive success and population structure vary in response to the climate variability across its range. We examined the reproductive success and stand structure of 10 Joshua tree populations distributed across the Mojave Desert. Joshua tree density varied by more than an order of magnitude across sites. At 8 of the 10 sites, nearly 80% of the Joshua trees were in bloom, and at the other two 40% were in bloom. The range of seed production and fruit set across the study populations varied by more than an order of magnitude. Fruit production occurred at all of our study sites suggesting that yucca moth pollinators were present at our sites. Increasing temperature had strong positive correlations with the number of trees in bloom (R^2^ = 0.42), inflorescences per tree (R^2^ = 0.37), and fruit mass (R^2^ = 0.77) and seed size (R^2^ = 0.89. In contrast, temperature was negatively correlated with Joshua tree stand density (R^2^ = -0.80). Positive correlations between temperature and greater flower and seed production suggest that warming may positively affect Joshua Tree reproduction while negative relationships between temperature and stand density are suggestive of potential constraints of warmer temperatures on establishment success.

## Introduction

Plants dependent on sexual reproduction require seed dispersal and germination, seedling establishment, recruitment and successful reproduction to maintain their populations. Success at each of these life stages are strongly influenced by the ability of plants to cope with abiotic stresses in their environment including climate extremes that can reduce vegetative growth and reproductive fitness and cause mortality [1, 2]. Biotic factors also play a prominent role in reproductive success and population structure including pollination, seed predation and dispersal, and herbivory [3–5]. The effects of climate extremes on plant populations vary dramatically depending on the magnitude, duration and timing of stress on vegetative and reproductive phases of the life cycle [6–8]. Since stresses in desert environments change dramatically over the course of the year, vegetative and reproductive phenology are critical in determining sensitivity to climate related stresses [9]. For example, plant functional processes that happen during cooler parts of the year may respond more positively to warming temperatures than processes that occur during the summer when high temperatures already limit plant function [2].

Climate has a foundational role in determining the structure and function of plant populations particularly in deserts where moisture limitation and temperature fluctuations are extreme. Soil moisture is the primary limiting factor for germination and establishment of desert plants [10] [11, 12]. Precipitation patterns in deserts of western North America have high annual and multi-year variability based on the Pacific Decadal Oscillation (PDO), El Nino Southern Oscillation (ENSO) and monsoonal patterns [13]. Climate change is expected to increase temperatures globally and increase variability in precipitation by altering the frequency and intensity of rain and drought events [14, 15]. For desert ecosystems of North America, current climate projections predict higher amounts of fall/winter precipitation, decreased spring/summer rain [16] and greater frequency of extreme weather events (drought and wet periods) [17]. It is critical that we understand how plant populations will respond to these changes in climate in order to predict range shifts of plant species under future climates.

Desert plants have developed a variety of adaptations to facilitate reproduction and survival in the face of temperature extremes and water deficit [18]. Perennial desert plants typically exhibit high stress tolerance that include maximizing water use efficiency and slower growth rates during periods of water and temperature stress but are able to readily utilize or store limiting resources (water and nutrients) as they become available in pulse events or during the cooler, wetter periods [2,19]. Fluctuations in temperature and precipitation across space and time makes understanding plant responses to climate complex in perennial desert plants.

Joshua tree (*Yucca brevifolia* Englm.) is a semi-succulent, arborescent evergreen. It is an iconic species in the southwestern US and its range is used to define the vast boundaries of the Mojave Desert. It is the largest plant in the Mojave Desert often exceeding 5 m in height and it is estimated that it can reach ages of 300 years [20]. Its complex vertical structure provides important habitat to birds, mammals and insects. Despite its prominence in plant communities of the Mojave Desert, surprisingly little has been published on its reproductive and structural ecology. The majority of research on Joshua tree has focused on its highly coevolved pollination relationship with the Yucca moth [21]. Outside its pollination biology only a few studies have been published on its reproductive ecology [5, 7, 22]. Based on observation it is known that population wide flowering events occur infrequently in Joshua tree. Its germination characteristics have been examined [7] but its population structure across its range is not well documented. These are critical knowledge gaps as research examining Joshua trees recent geological history suggest that its range has contracted significantly in the last 10,000 years and climate forecasting suggest that widespread population losses may occur in response to climate change [23].

In 2013, a widespread Joshua tree flowering event provided a unique opportunity to study the reproductive ecology of this unique species. In parallel we assessed population structure of Joshua tree forests, for the first time, across it range in the Mojave Desert. The objective of this study was to characterize flower and fruit set and population structure of Joshua tree across its range and identify how these characteristics varied due to variation in climate (temperature and precipitation) across the Mojave Desert. We hypothesized that flowering and fruit set, which are initiated in the cooler winter/spring seasons would be positively correlated to warmer temperatures, but that populations density would be negatively correlated with temperature perhaps due to reduced establishment and survival with warmer temperatures during the summer months.

## Materials and methods

### Study sites and experimental design

**Fig 1:**
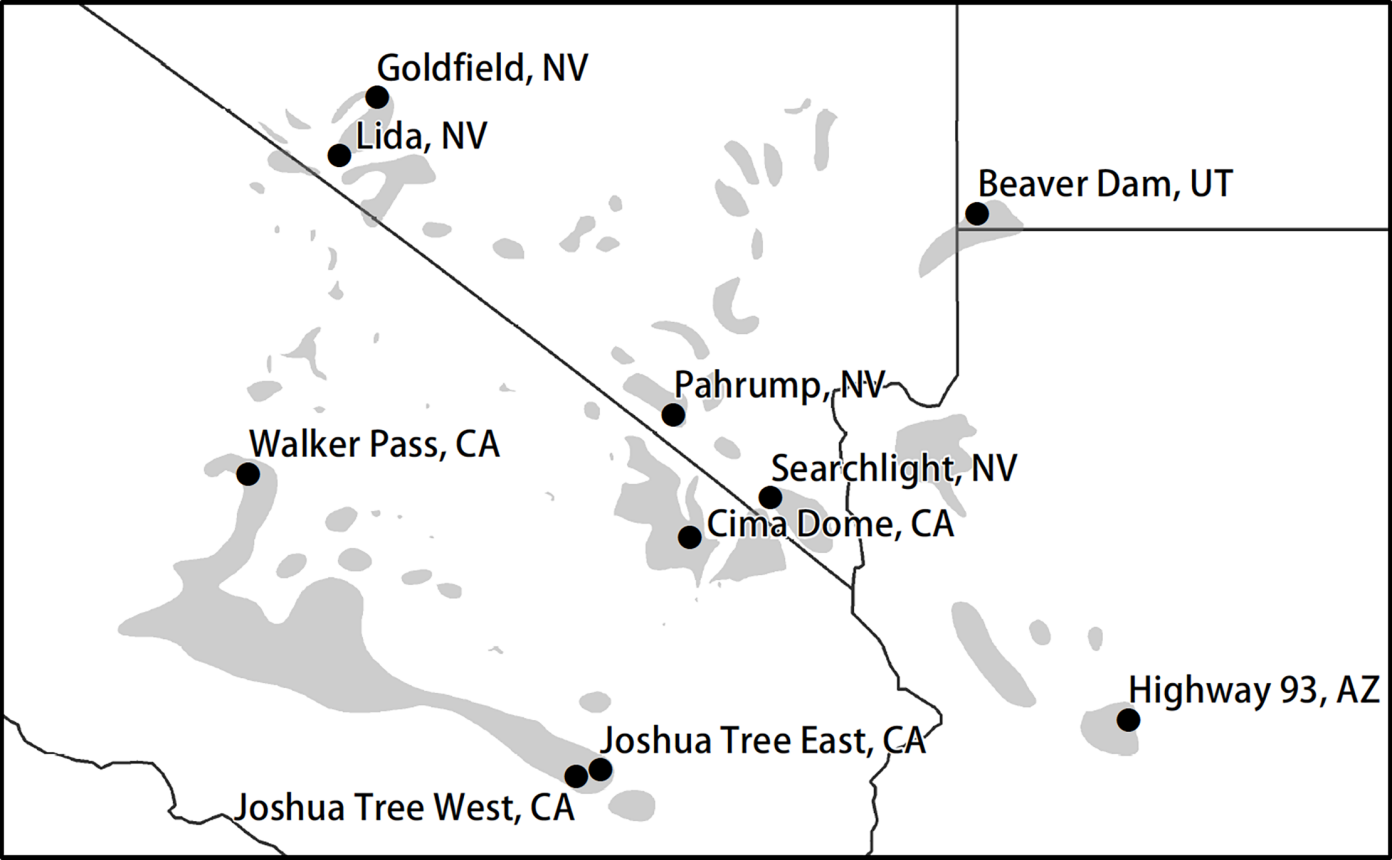
Map of Joshua tree’s range in the southwestern US (gray polygons) and our 10 study locations across the range of that distribution. Data for the mapping the distribution of Joshua tree was obtained from the following database: https://databasin.org/datasets/b74f96cc008d4c7398ea0ef0bb6b4078.

To characterize Joshua tree across its range, ten study sites were selected across four states (California, Utah, Nevada and Arizona) that span the geographical and elevational range of Joshua tree (Fig 1). Site selection in our study maximized coverage across Joshua tree’s range but was done without any information about flowering at specific sites to avoid skewing our survey. Sites were visited between May and June 2013. At each study site, we ran six 100 meter transects spaced at 1 km intervals to get a robust measure of population characteristics and reproduction.

### Measurements of population structure and reproduction

Measurements were made on 120 Joshua trees per site (1200 total trees). At each 10-meter increment point along each transect, we measured the distances to the two nearest Joshua trees on each side of the transect tape for estimates of population density using point distance methods according to Diggle [24]. On these same trees (20 trees/transect) we assessed tree height, trunk diameter and flower and fruit production. Tree height was determined using a telescoping meter stick. Trunk diameter was measured 20 cm above the soil surface using tree calipers. Branches, inflorescences and total fruits were counted on each tree. Percent of trees in bloom was calculated as the number of trees with at least 1 inflorescence relative to the total number of trees surveyed. The number of inflorescences produced were counted for each tree to calculate the average number of inflorescences produced per tree along each transect. A single fully developed fruit was collected from each tree for measurements of fruit mass and seed number (120 per site, 1200 total). Authorities that provided approval of fruit collection were: Josh Hoines (Joshua Tree National Park, California); Debra Hughson (Cima Dome, California); Cody Carter (Highway 93 Arizona), Dawna Ferris (Beaver Dam, Utah), Laura Kobelt (Pahrump and Searchlight, Nevada), Dennis Kearns (Walker Pass, California) and Anna Obrien (Goldfield and Lida, Nevada). Fruits were placed in paper bags, and dried at 40 C for three weeks. Dried fruits were measured for total mass and then total seeds per fruit were counted, and measured for mass using a balance. The total number of fruits counted along each transect and the number of seeds counted were divided by the number of trees along each transect to calculate the number of fruits per tree and seeds per tree. Seed production per unit ground area was estimated based on number of seeds per fruit, number of fruits per tree and number of trees per hectare but recalculated on a per m^-2^ basis.

### Climate data

Precipitation and temperature data for each study site from 1980–2013 was used to calculate the following climate variables: annual temperature maximum (hottest temperature of the year averaged across the 30 year period); annual temperature minimum (coldest temperature of the year averaged across the 30 year period), monthly temperature maximum (averaged daily maximum of the hottest month of the year averaged across the 30 year period), monthly temperature minimum (mean daily minimum of the coldest month of the year averaged over the 30 year period), 30-year temperature average (mean annual temperature averaged over the 30 period), 30-year monthly precipitation maximum (sum of precipitation for wettest month of each year, averaged over the 30 year period), monthly precipitation minimum (sum of precipitation for driest month of each year, averaged over the 30 year period) and the 30-year precipitation average (annual summed precipitation averaged over the 30 period). The average 30 year precipitation total for March and April (spring) and September and October (fall) were estimated to examine the effects of variation in spring and fall precipitation [16, 17]. A 30-year climate window was chosen to look at the long term climate average experienced by this long-lived species [20]. All climate data was obtained from PRISM Climate Group, Oregon State University, http://prism.oregonstate.edu, created 7 December 2016.

### Statistical analysis

Data exploration was conducted according to the methods of Zuur [25]to test that all model assumptions were met. All response variables met equal variance assumptions. We tested multicollinearity among and between precipitation and temperature measurements using Pearson’s correlation coefficient. Thirty year average, minimum, and maximum air temperatures were highly correlated (> 0.8 r^2^). Thirty year mean maximum and minimum temperatures were not highly correlated with thirty-year average, minimum, and maximum air temperatures (< 0.5 r^2^). There were no problems with multicollinearity between precipitation and temperature variables. We used model selection to identify best approximating models that predicted which climate variables best explained variation in Joshua tree reproduction and stand structure. We did this by competing each climate variable in linear regression models against one another for each response variable. The best model for each response variable was based on minimization of Akaike Information Criterion value corrected for sample size (i.e., AICc; [26]. AICc analysis was conducted in R using the MuMIn package [27, 28]. Competing models were kept simple (K ≤ 3) in order to avoid overfitting [26]. All models were better than the null model.

In each case the best model (all other models 2 >ΔAIC) included a single climate variable. Because of the simplicity of the models the strength and direction of estimates of the identified climate variable for each response variable was modeled with simple linear regression using JMP software (version 13). Tukey’s tests were used for multiple pairwise comparisons also using JMP software.

## Results

### Variation in stand structure across sites

**Fig 2:**
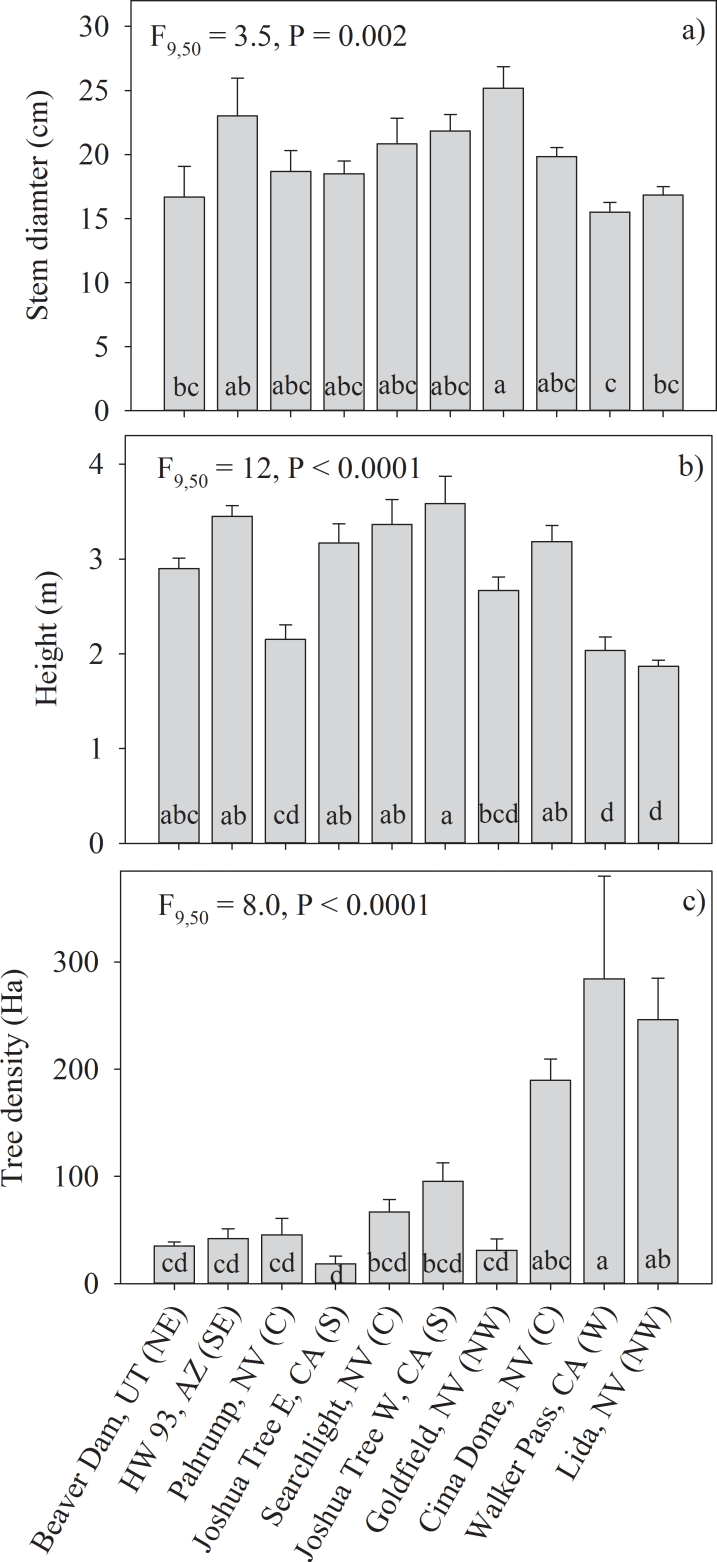
Recruitment patterns and stand structural characteristics of at the 10 study sites. Mean values are calculated from n = 6 transects per site and presented with ± 1 SE. F-statistics and P-values presented that test the significance of variability in population characteristics across the 10 study sites. Sites are ordered from hottest to coolest from left to right. Letters in parentheses behind each site represent range location: northeast (NE), northwest (NW), southeast (SE), central (C), south (S), west (W), Multiple mean comparisons conducted using Tukey’s HSD with different letters between sites denoting significant mean differences.

Joshua tree density, height, and trunk diameter varied significantly across the 10 study sites (Fig 2). Joshua tree trunk diameter and tree height varied less than 2-fold across study sites (Fig 2A and 2B). The range of Joshua tree population density varied by more than an order of magnitude from 20 trees per hectare at Joshua Tree East to 280 trees per hectare at Walker Pass (Fig 2C). Walker Pass, Lida and Cima Dome had Joshua tree stand densities that were several-fold greater than the other 7 sites.

### Variation in reproductive ecology across sites

**Fig 3:**
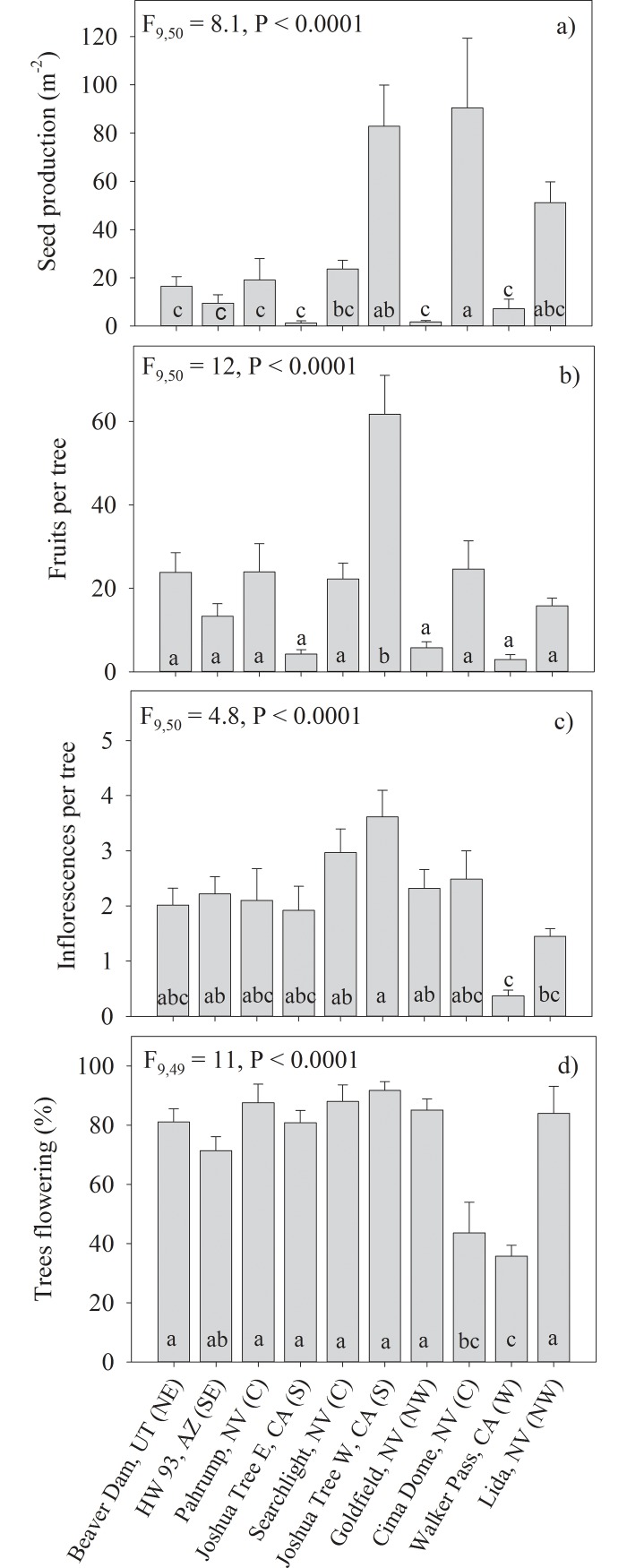
Variation in reproductive characteristics across the 10 study sites. Mean values presented with ± 1 SE. F-statistics and P-values presented that test the significance of variability in reproductive characteristics across the 10 study sites. Sites are ordered from hottest to coolest from left to right. Letters in parentheses behind each site represent range location: northeast (NE), northwest (NW), southeast (SE), central (C), south (S), west (W), Multiple mean comparisons conducted using Tukey’s HSD with different letters between sites denoting significant mean differences.

Flowering, fruit set and seed production varied strongly across the 10 study sites. The range of seed production and fruit set across the study sites varied by more than an order of magnitude (Fig 3A and 3B). Joshua tree bloomed strongly across its range in 2013 with most sites averaging close to 80% trees in bloom, with the only anomalies being Cima Dome and Walker Pass, which were closer to 40% (P <0.0001). The percent of trees in bloom and the number of inflorescences produced per tree were much less variable across sites. The percent of flowering trees and inflorescences produced per tree were highest at Joshua Tree West (P <0.0001) and lowest at Walker Pass (P <0.0001) and did not vary more than 2-fold among the other 8 sites (Fig 3C and 3D). There was a strong positive correlation between inflorescences per tree and fruits per tree (r = 0.74, P < 0.0001).

### Relationship between Joshua tree population structure, reproduction and climate variability

**Fig 4:**
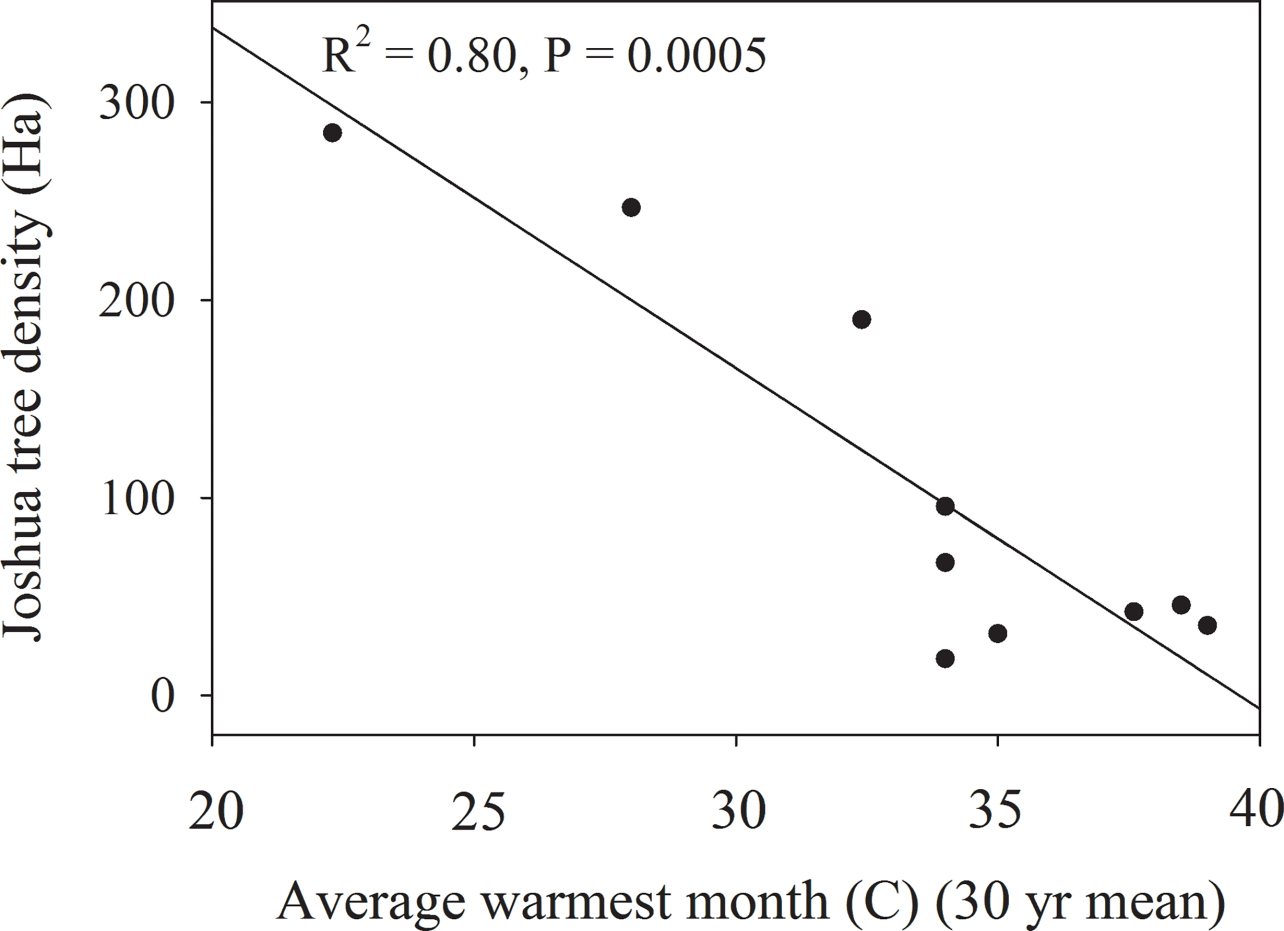
Relationship between average warmest month temperature and stand characteristics across the 10 study sites using regression analysis.

**Fig 5:**
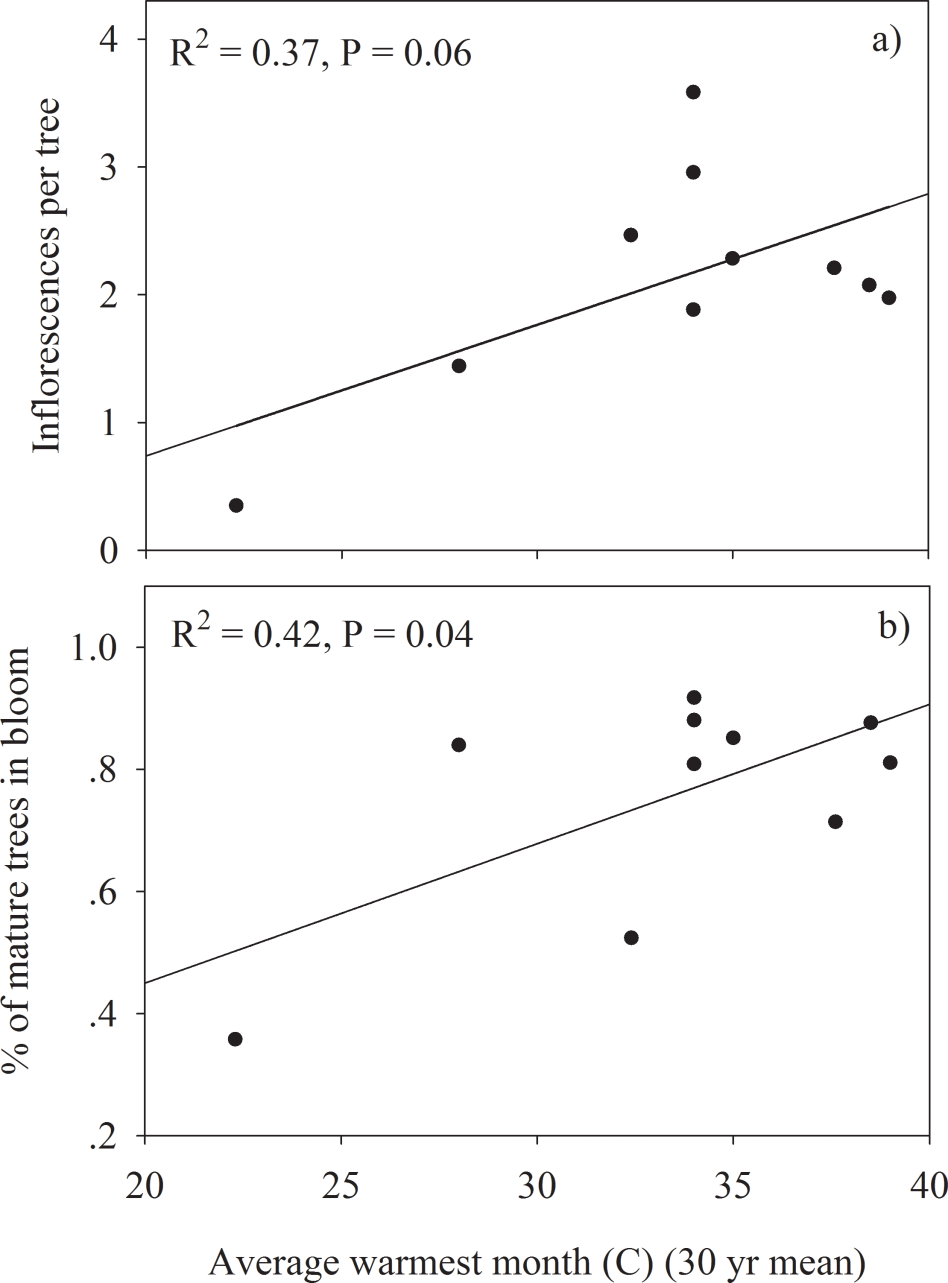
Relationship between 30-year temperature average and fruit and seed mass across the 10 study sites using regression analysis.

**Fig 6:**
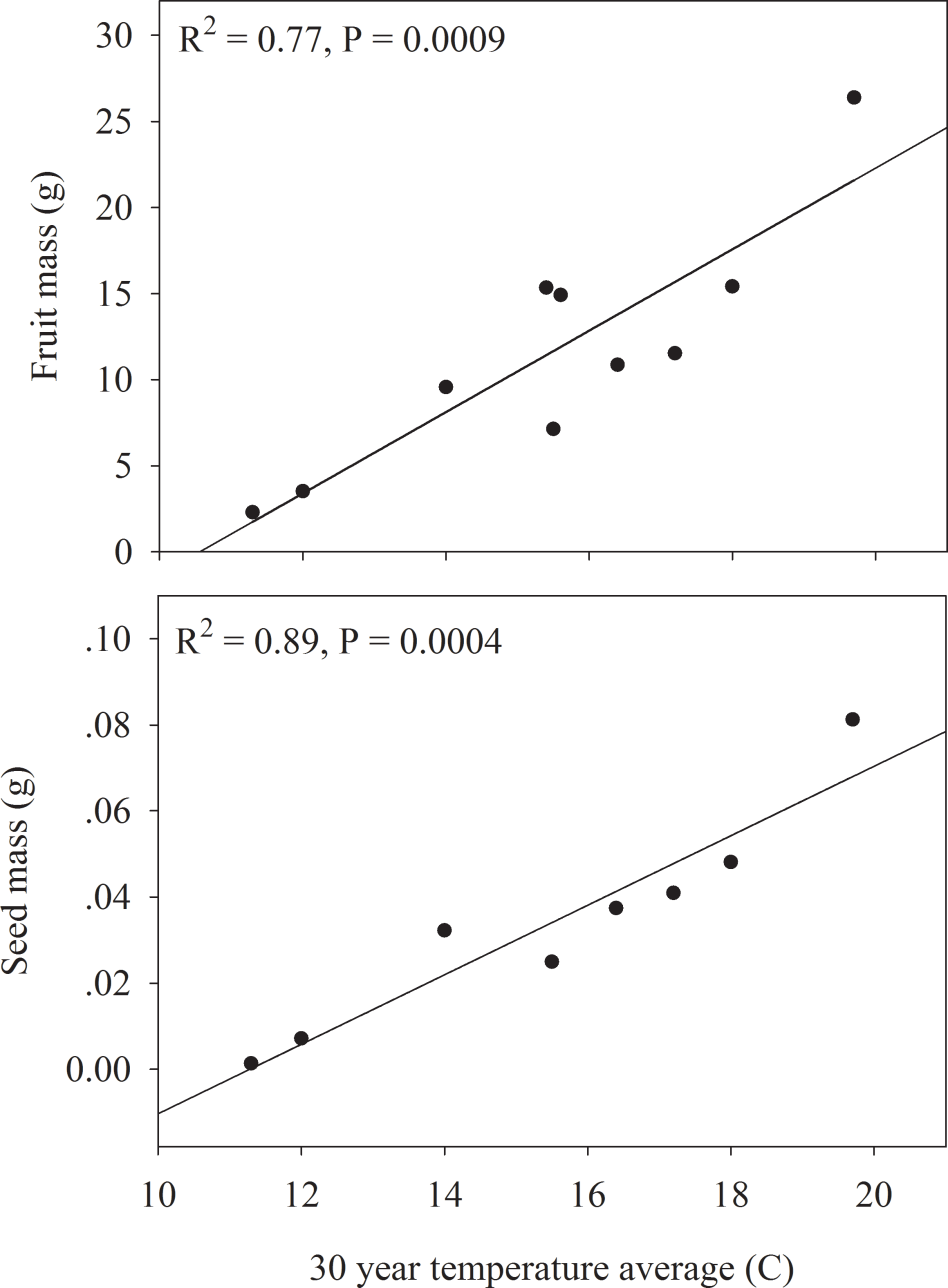
Relationship between 30-year temperature average and fruit and seed mass across the 10 study sites using regression analysis.

Joshua tree density was most strongly correlated with the 30 year average monthly temperature maximum at each site (R^2^ = -0.80) (Fig 4). Stand tree and trunk diameter were not strongly correlated with any of the climate variables (Table 1). Reproductive success of Joshua tree populations across the Mojave Desert was most strongly correlated with temperature. Monthly temperature maximum at each site was positive correlated with number of trees in bloom (R^2^ = 0.42) and inflorescences per tree (R^2^ = 0.37) (Fig 5A and 5B). The 30-year temperature average at each site showed strong positive correlations with fruit mass (R^2^ = 0.77) and seed mass (R^2^ = 0.89) (Fig 6A and 6B). The 30-year precipitation average, and the 30-year annual maximum and minimum temperatures were also significantly correlated with fruit mass and seed mass but not as strongly as the 30-year temperature average (Table 1). The number of fruits per tree, seeds per tree and seeds produced per m^2^ of ground area were not significantly correlated with precipitation or temperature variables (Table 1).

**Table 1:** Pearson correlation coefficients indicating the direction and the significance of the relationships between climate variables and stand structure and reproduction of Joshua tree across the 10 study sites.

	Tree density	Tree height	% flowering trees	Inflorescences/tree	Fruits/tree	Fruit mass	Seed mass	Seeds/tree	Seeds m2
Tm annual max	**-0.52**	0.33	-0.06	0.04	0.05	**0.72**	**0.78**	0.11	-0.42
Tm annual min	-0.1	0.2	-0.35	-0.09	0.06	**0.57**	**0.64**	0.13	-0.22
Tm monthly max	**-0.89**	0.49	**0.64**	**0.61**	0.27	0.52	**0.65**	0.37	-0.11
Tm monthly min	0.04	0.28	-0.32	0.02	0.23	0.4	0.16	0.27	0.04
Tm 30 year average	-0.4	0.39	-0.01	0.08	0.13	**0.87**	**0.94**	0.21	-0.26
Precip month max	0.25	0.47	-0.44	0.05	0.06	0.58	0.56	0.09	0.35
Precip monthly min	0.29	-0.35	0.12	-0.16	-0.2	-0.44	-0.44	-0.21	0.14
Precip spring 30 yr average	0.07	0.42	0.07	0.06	0.03	0.55	0.47	0.01	0.19
Precip fall 30 yr average	0.1	0.51	0.1	0.06	0.16	0.64	0.56	0.13	0.14
Precip 30 year average	0.04	0.39	-0.1	0.11	0	**0.59**	**0.67**	0.04	0.17

## Discussion

One anticipated consequence of climate change is range contraction of sensitive and ecologically important species whose loss significantly alters the habitat conditions and function of ecosystems. Desert flora, can be sensitive to climate change due to the moisture and temperature extremes of deserts. Joshua tree forests are an ecologically important vegetation type in the Mojave Desert and have potential for range contraction in response to climate change although the mechanisms of contraction are not well understood [23]. In fact, for such an iconic species it is surprising how little research has been done on the most basic parts of Joshua tree biology and ecology. Our analysis of 10 Joshua tree populations with broad coverage across its range shows for the first time strong variation in reproductive success and stand structure that is tightly linked to variability in temperature across the Mojave Desert. Our hypothesis that warmer temperatures would be positively correlated with reproduction but negatively correlated with population density was generally supported by the data. Flower production, and fruit and seed mass were all positively correlated with warmer temperatures (Figs 5 and 6), while stand density was negatively related to higher temperatures (Fig 4). While correlation analysis suggests a role for temperature in influencing Joshua tree reproduction and stand density, controlled studies are needed to test any causal relationship between temperature and Joshua tree reproduction and establishment.

### Stand structure

Joshua tree density across the 10 study sites was much more variable than Joshua tree height or stem diameter (Fig 2). Germination and establishment success of Joshua tree is influenced by several environmental factors that may have contributed to the observed variation in stand density differences across sites [7]. It has been documented that Joshua tree establishment is often facilitated by woody shrubs and grasses so variation in shrub abundance and cover can affect recruitment success [29, 30]. Temperature extremes [31] [32], drought, and fire [1] have been shown to affect germination, establishment and survival of Joshua tree. The abundance and diversity of rodent populations, which can vary significantly depending on site conditions in the Mojave Desert [33, 34] strongly regulates the dispersal of Joshua tree seeds and can result in high levels of seed predation [22, 35]. Rodent herbivory in some instances can lead to mortality of adult Joshua trees [36] [37]. The negative correlations between temperature and spatial variation of Joshua tree density (Fig 4A) may be affected by a combination of the negative effects of warmer temperatures on seed germination, recruitment and drought mortality of large Joshua trees that are more likely to occur under warmer conditions [1].

### Reproductive success

Joshua tree flowering occurs episodically and widespread blooming events are thought to be rare although published studies are lacking. Reports of widespread blooming of Joshua tree from various locations across the Mojave Desert in the early spring of 2013 provided an opportunity to study patterns of Joshua tree reproduction across its range. Our analysis confirmed that the blooming event of 2013 occurred across the sampled range of Joshua tree forests in our study with significant percent of trees blooming at all of our study sites (Fig 2). While traveling across the Mojave Desert to survey the target populations we observed many more Joshua tree populations along the way and observed that they too were strongly in bloom (personal observation: Sam St. Clair, Josh Hones). Interestingly, we also observed a high proportion of blooming Joshua trees in urban environments of St. George Utah and Phoenix Arizona in irrigated landscapes, 80+ kilometers outside of the range where they naturally occur. A survey of fifty Joshua trees located in the median of Interstate-15 just north of St. George, Utah showed that 86% were in bloom, which is very similar to the blooming rates we saw for most of the natural populations (Fig 3). These observations along with less than 50% of variation in Joshua tree flowering being explained by climate variability (Table 1, Fig 5) suggest that non-climate related factors may also contribute to the coordination of range wide Joshua tree blooming events.

Yucca fruits only develop when pollination by obligate yucca moths is successful [5]. Fruit production was positively correlated with inflorescence production (r = 0.74) suggesting that flower production not pollination was the key limitation to fruit production. While there was high variability in fruit production across sites, fruits were produced at all of our study locations (Fig 3B). Because the Joshua tree-yucca moth pollination system is obligate [21] the presence of fruits indicate there are active yucca moth populations performing pollination services across Joshua trees present range. Regional collapses of yucca moth populations have led to complete failure of fruit production in the closely related *Yucca baccatta* in the Mojave Desert [5]. Monitoring the effects of climate change on Joshua tree reproduction into the future should be done in the context of the status of their obligate yucca moth pollinators.

The number of blooming trees and inflorescences per tree, and fruit and seed mass all showed strong positive correlations with long-term temperature averages and weak relationships with precipitation in our analysis (Figs 5 and 6). Winter minimum temperatures may be a bigger constraint to reproductive success than summer maximum temperatures or water availability since Joshua tree reproduction happens in the spring period [19]. It is estimated that 80% of the yearly uptake of CO_2_ through photosynthesis occurs during the winter-spring period when minimum temperatures are more limiting to its physiology than water availability [19].

## Conclusions

From our data there is clear evidence of blooming and fruit production across Joshua trees range in 2013 although we still don’t understand the environment cues that lead to large bloom events in Joshua tree [38]. It is interesting to note the positive responses of Joshua tree reproduction to warmer temperatures in contrast to the negative relationship of warmer temperatures on stand density (Figs 4–6). These correlations suggest that even if there are positive effects of warmer temperatures on reproductive potential of Joshua tree, they may be offset if there are negative effects of warmer temperature on stand density [1,7].

## Supporting information

S1 File Minimum data set. (XLSX)
